# Active Silicone Oil Removal with a Transconjunctival Sutureless System: Is the 23-Gauge System Safe and Effective?

**DOI:** 10.4274/tjo.15807

**Published:** 2016-01-05

**Authors:** Mahmut Kaya, Ayhan Özyurt, Arif Taylan Öztürk, Duygu Er, Süleyman Kaynak, Nilüfer Koçak

**Affiliations:** 1 Dokuz Eylül University Faculty of Medicine, Department of Ophthalmology, İzmir, Turkey

**Keywords:** 23-gauge transconjunctival sutureless technique, silicone oil removal

## Abstract

**Objectives:**

To evaluate the safety and efficacy of active silicone oil removal with a 23-gauge (G) transconjunctival sutureless system.

**Materials and Methods:**

One hundred sixteen eyes of 113 patients who had previous retinal detachment surgery with pars plana vitrectomy and silicone oil injection surgery, and underwent silicone oil removal surgery with 23-G transconjonctival sutureless technique in our clinic between January 2009 and April 2014 were reviewed retrospectively. The patients were evaluated with regard to postoperative changes in best corrected visual acuity and intraocular pressure (IOP), and complications that occurred during and after surgery.

**Results:**

Of the 113 patients with mean age of 61.1±9.7 years (29-88 years), 62 (54.8%) were males and 51 (45.2%) were females. Silicone oil removal was performed 8.43±5.24 months after the initial surgery. Mean follow-up was 13.38±4.35 months. Visual acuity improved in 69 eyes (59.48%). Anatomic success was achieved in 113 eyes (97.41%). Mean IOP was 16.2±7.2 mmHg at baseline and 14.4±2.6 mmHg at postoperative day 1 (p=0.643). Eight eyes needed suturation of at least one sclerotomy. Retinal redetachment occurred in 3 eyes (2.5%) resulting in a decrease in vision. There were no cases of choroidal detachment, endophthalmitis, clinically significant corneal endothelial decompensation, or macular edema.

**Conclusion:**

Active removal of 1,300-centistoke silicone oil with a 23-G transconjunctival sutureless system is a simple, sutureless technique causing minor surgical trauma. Active removal of silicone oil with the 23-G transconjunctival sutureless technique was found to be effective and safe in both phakic and pseudophakic eyes.

## INTRODUCTION

Silicone oil is currently used as a replacement for the vitreous during vitreoretinal surgeries, especially in complicated conditions such as retinal detachment (RD) with giant tears, proliferative vitreoretinopathy, trauma, and endophthalmitis.1 Because the long-term presence of silicone oil in the eye leads to complications such as cataract, glaucoma, late corneal decompensation, and band keratopathy, it is recommended that silicone oil be removed as soon as possible after it has fulfilled its purpose as a tamponade.^1,2,3^

Silicone oil removal (SOR) is an important procedure in vitreoretinal surgery. Although there are various methods and techniques for SOR,^4,5,6,7^ the procedure can basically be performed through either the anterior or the posterior segment.8 SOR via the anterior segment is preferred in aphakic patients or with cataract surgery, whereas removal via the pars plana is preferred in phakic and pseudophakic patients.8 SOR through the pars plana also allows adequate examination of the retina during procedures like epiretinal membrane peeling, endolaser application, membranectomy and posterior capsulotomy.

SOR can be performed with 20-gauge (G), 23-G or 25-G extraction cannulae.^9,10,11^ In recent years, smaller gauge sclerotomies have become a preferred technique in SOR.^9,10,11^ The main advantages of small-gauge sclerotomies are their self-sealing properties, early incision healing, low ocular surface irritation and more cosmetically satisfying results.^10,11,12^ The major benefit of using 23-G instruments versus 25-G instruments during surgical procedures is that they are more powerful and self-sealing (valve system).

The purpose of this study was to evaluate the effectiveness and safety of the 23-G transconjunctival sutureless system in SOR.

## MATERIALS AND METHODS

The study included 116 eyes of 113 patients who were diagnosed with RD (rhegmatogenous RD associated with multiple or giant tears, diabetic tractional RD, traumatic RD, etc.), underwent pars plana vitrectomy (PPV) with silicone oil injection, and later underwent SOR by 23-G transconjunctival sutureless technique in the retina unit of our clinic between January 2009 and April 2014. All patients received 1,300-centistoke (cSt) silicone oil tamponade. Data from the patient’s medical records were reviewed retrospectively. Patients’ predisposing factors for RD prior to PPV, type of RD, presence or absence of macular involvement, RD duration, best corrected visual acuity (BCVA) and lens status were recorded. We included patients over 18 years of age and excluded patients with retinal redetachment, hypotonic eyes [intraocular pressure (IOP) <8 mmHg], or scleral thinning in the sclerotomy area prior to SOR. Data regarding patients’ BCVA (ETDRS chart), slit-lamp examination, IOP measured by Goldmann applanation tonometry, macular status on spectral domain optic coherence tomography (SD-OCT), and dilated fundus examination after SOR were recorded for all patients. There were no complications related to silicone oil such as glaucoma or keratopathy in these eyes. Complete retinal reattachment at 6 month follow-up was considered anatomic success; a BCVA improvement of ≥1 LogMAR row was considered functional success.

## Surgical Technique

Before surgery, retrobulbar anesthesia (2 ml lidocaine+2 ml bupivacaine) was administered to all patients using a standard 4 ml Atkinson needle. A pars plana entry was performed with the Constellation vitrectomy system (Alcon laboratories, USA) using 23-G vitrectomy system and valved trocars in the inferotemporal area and superior quadrant at 4 mm from the limbus in phakic patients and 3.5 mm in pseudophakic patients. An infusion port was inserted in the temporal area and the fluid pressure was set to 25 mmHg, after which the silicone oil was removed as a whole from the superior quadrant with 480-650 mmHg vacuum. After the bulk of the silicone oil was removed, the remaining emulsified particles were cleared. For patients with emulsified silicone particles in the anterior chamber, an incision into the anterior chamber was made with a 20-G Stiletto blade and the emulsified particles were removed by passive aspiration. Because complete retinal reattachment was observed on preoperative dilated fundus examination and additional laser treatment was deemed unnecessary, there was no further entry into the eye after SOR. Following SOR, the trocars were removed and leakage was tested. In case of leakage, the sclerotomy was sutured with 8/0 vicryl suture.

Statistical Package for the Social Sciences version 17.0 software was used for statistical analyses. The mean and standard deviation were calculated for all data. Statistical analysis was done with a parametric t-test. Dependent samples t-test was used to evaluate changes in BCVA and IOP before and after SOR. Level of significance was accepted as α=0.05.

## RESULTS

One hundred sixteen eyes of 113 patients were included in the study. The mean age of the patients was 61.1±9.7 years (range, 29-88 years); 62 (54.8%) were male, 51 (45.2%) were female. Sixty-one (52.5%) of the eyes were left eyes. Fifty-six (48.2%) of the eyes were pseudophakic. In pseudophakic patients, the mean time between cataract surgery and RD was 3.6±2.5 years (median, 4 years; range, 8 months-9 years). The mean duration of follow-up after SOR was 13.38±4.35 months (range, 6-26 months). The etiologies of the patients’ RD are shown in Table 1.

The clinical characteristics of the patients are summarized in Table 2. The mean time between silicone oil injection during vitrectomy surgery and SOR using the 23-G transconjunctival sutureless technique was 8.43±5.24 months (range, 3-16 months). The mean BCVA at the last examination before SOR was 1.31±0.85 logMAR (range, 0.5-3.0 logMAR). At 3 months after SOR, the mean BCVA was 0.84±0.74 logMAR (range, 0.05-3.0 logMAR). The improvement in BCVA after SOR was statistically significant (p<0.001). Of the 40 phakic eyes, 18 (45%) developed cataract associated with silicone oil. The mean IOP was 18.2±7.1 mmHg (range, 12-34 mmHg) before SOR and 16.4±2.6 mmHg (range, 12-26 mmHg) after SOR (p=0.643). Prior to SOR, an elevated IOP of 28-34 mmHg was detected in 16 (13.8%) of the 116 eyes, and was controlled in all cases with a single fixed combination (10 mg/ml brinzolamide+5 mg/ml timolol or 20 mg/ml dorzolamide HCL+5 mg/ml timolol maleate) anti-glaucoma therapy. None of the patients exhibited elevated IOP requiring medical treatment after SOR. Silicone oil was found in the anterior chambers of 13 of the pseudophakic eyes. In these patients, the silicone oil in the anterior chamber was successfully removed in the same surgical session as the SOR from the posterior segment. Retinal redetachment occurred in 3/116 (2.5%) eyes within 3 months of SOR. The first of these cases had giant tear RD, and SOR was performed in the 8th postoperative month; the second and third cases had RD due to multiple tears and tractional RD, respectively, and both underwent SOR in the 6th postoperative month. All three of the eyes had successful retinal attachment with vitrectomy surgery (using silicone oil tamponade).

In our study, anatomic success was achieved in 113/116 (97.41%) eyes in the first vitrectomy surgery using silicone oil tamponade. Functional success was achieved in 88/116 (75.86%) eyes after phacoemulsification of the eyes that developed cataract. Eight (6.89%) eyes exhibited leakage after trocar removal and required suturing. None of the patients developed persistent hypotony following the 23-G transconjunctival sutureless technique.

## DISCUSSION

In recent years, sutureless small-gauge incisions have been preferred for the majority of intraocular surgeries in order to provide better anatomic outcome and functional improvement. The development of small-gauge (23-G and 25-G) vitrectomy, or minimally invasive vitreous surgery, without compromising outcomes is one of the most important goals of surgeons.^13^ With this aim, Fujii et al.^6^ began to use the 25-G transconjunctival sutureless vitrectomy technique in the surgical treatment of uncomplicated vitreoretinal patients. In later years, Eckardt^7^ introduced the 23-G transconjunctival sutureless vitrectomy technique. Surgeons familiar with 20-G preferred 23-G transconjunctival sutureless vitrectomy over the 25-G technique due to the greater durability of 23-G instruments. In current conditions, however, 25-G vitrectomy is preferred when possible. These developments in vitrectomy surgery have led to innovations in other surgeries related to the posterior segment. The main benefits of small incisions are that they generally do not require suturing, they prevent adhesions from repeated operations, and they provide a short operating time, minimal trauma, less irritation, comfortable surgery and speedy recovery.^9,10,11,12^

There are currently several different methods (20-G, 23-G and 25-G) by which silicone oil can be removed.^10,11,12^ SOR by 25-G microcannula was first described by Kapran and Acar.^10^ Later, it was observed that the eye was more stable and the procedure was more comfortable when silicone oil was removed using a 23-G transconjunctival system compared to the 20-G system.^12,14^ In 20-G systems, a tendency to collapse was observed due to incision with a 20-G MVR blade. Although 23-G vitrectomy systems are considered simple and reliable, there have also been reports of possible disadvantages such as intraoperative subconjunctival hemorrhage, incision leakage, trocar displacement, and leakage of infusion fluid under the conjunctiva; in fact, in some cases it was necessary to continue the surgery with a 20-G system.^11,15^

The most common problem encountered in sutureless systems is hypotony.^16,17^ In our study, IOP was compared before and 1 day after SOR. Although a drop in IOP was observed, the difference was not statistically significant. Similarly, Kapran and Acar^10^ investigated IOP changes after SOR with a 25-G system; although they observed a significant drop in IOP 2 hours after SOR, no significant differences were found at postoperative 1 day, 1 week or 1 month. Reports vary regarding the need for postoperative sutures in small-gauge transconjunctival vitrectomy systems. In the current study, 6.8% of the eyes had positive leakage tests after SOR and required suturation of at least one of the sclerotomy sites. Doğanay et al.^11^ used a 23-G vitrectomy system for SOR in 34 patients and sutured at least one of the sclerotomy sites in 15 (44%) cases. In a study by Patwardhan et al.^9^ after SOR from 20 eyes with a 23-G system, suturation was required in 3 (15%) eyes. Romano et al.^16^ sutured at least one sclerotomy site of 5 (50%) of 10 eyes after SOR with a 23-G transconjunctival system. In contrast, Kapran and Acar^10^ reported that none of the 13 eyes in their study required suturation after SOR with a 25-G transconjunctival sutureless system.

The most significant risk in sutureless surgeries is development of endophthalmitis.^18,19,20^ In the current study, endophthalmitis did not occur in any of the 116 eyes of 113 patients that underwent SOR via 23-G transconjunctival sutureless system. In all of our patients, 5% povidone-iodine drops were instilled and the eyelids and face were cleaned with 10% povidone-iodine preoperatively. During surgery, the conjunctiva was held away from the trocar entry point and the trocars were inserted gradually into the sclera. At the end of the procedure, the edges of the scleral incisions were patted down over the entry point and closed with the removed intact conjunctiva and Tenon’s capsule.

Retinal redetachment after SOR has been reported at rates of 6-33%.^21,22,23^ Kapran and Acar^10^ observed it in 2 cases (15.3%); Doğanay et al.^11^ in 2 cases (5.8%). In our study, retinal redetachment was found in 3 eyes (2.5%). In all 3 eyes, reattachment was successfully achieved with a second surgery. Romano et al.^16^ reported that none of their patients developed retinal redetachment.

In the current study, all patients with the exception of the three with retinal redetachment had improved or stable visual acuity. There was a statistically significant increase in mean BCVA following SOR. We achieved anatomic and functional success rates of 97.4% and 75.8%, respectively. Of the phakic eyes, 45% developed cataract at different times. Cataract surgery was not performed in the same surgical session as the SOR.

## CONCLUSION

Our study demonstrates that the 23-G transconjunctival sutureless technique for SOR has no negative impact on anatomic or functional success, and considering the benefits provided, it was concluded to be a reliable and effective system. Using transconjunctival small-gauge sclerotomy technique in SOR provides the advantages of a closed system as well as benefits such as protection of the conjunctiva, shortened surgery time and fewer suture-related complications. We believe that with the development of new techniques and methods, the 23-G transconjunctival sutureless system will be safe to use in SOR regardless of phakic, pseudophakic or aphakic lens status.

## Ethics

Ethics Committee Approval: None (retrospective study), Informed Consent: Patients were informed of the details of the study and all signed an informed consent form prior to their participation.

Peer-review: Externally peer-reviewed.

## Figures and Tables

**Table 1:**
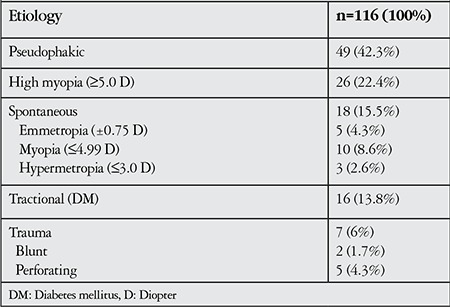
Causes of retinal detachment

**Table 2:**
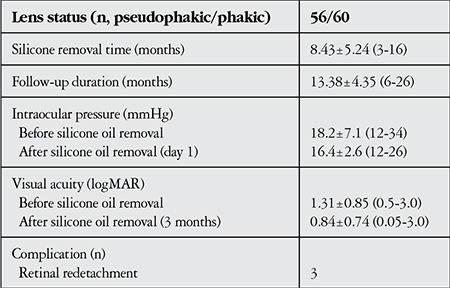
Clinical characteristics of the patients
